# Genome-wide identification and analysis of the *NLR* gene family in *Medicago ruthenica*

**DOI:** 10.3389/fgene.2022.1088763

**Published:** 2023-01-10

**Authors:** Chunyan Tong, Yutong Zhang, Fengling Shi

**Affiliations:** ^1^ College of Grassland, Resources and Environment, Inner Mongolia Agricultural University, Hohhot, China; ^2^ Key Laboratory of Grassland Resources (IMAU), Ministry of Education, Hohhot, China

**Keywords:** *Medicago ruthenica*, *NLR* gene family, chromosome distribution, phylogenetic analysis, syntenic analysis, expression analysis

## Abstract

*Medicago ruthenica*, important forage in the legume family, possesses high nutritional value and carries abundant tolerance genes. This study used whole-genome data of *M. ruthenica* to perform a genome-wide analysis of the nucleotide-binding site-leucine-rich repeat receptor (*NLR*) gene family, which is the largest family of plant disease resistance genes (*R* genes). A total of 338 *NLR* genes were identified in the *M. ruthenica* genome, including 160 typical genes that contained 80 coiled-coil (CC)-NBS-LRR (*CNL*) genes, 76 toll/interleukin-1 receptor (TIR)-NBS-LRR (*TNL*) genes, four resistance to powdery mildew 8 (RPW8)-NBS-LRR (*RNL*) subclass genes, and 178 atypical *NLR* genes encoding proteins without at least one important domain. Among its eight chromosomes, *M. ruthenica* chromosomes 3 and 8 contained most of the *NLR* genes. More than 40% of all *NLR* genes were located on these two chromosomes, mainly in multigene clusters. The *NLR* proteins of *M. ruthenica* had six highly conserved motifs: P-loop, GLPL, RNBS-D, kinase-2, RNBS-C, and MHDV. Phylogenetic analysis revealed that the *NLR* genes of *M. ruthenica* formed three deeply separated clades according to the N-terminal domain of the proteins encoded by these genes. Gene duplication and syntenic analysis suggested four gene duplication types in the *NLR* genes of *M. ruthenica*, namely, tandem, proximal, dispersed, and segmental duplicates, which involved 189, 49, 59, and 41 genes, respectively. A total of 41 segmental duplication genes formed 23 *NLR* gene pairs located on syntenic chromosomal blocks mainly between chromosomes 6 and 7. In addition, syntenic analysis between *M. truncatula* and *M. ruthenica* revealed 193 gene pairs located on syntenic chromosomal blocks of the two species. The expression analysis of *M. ruthenica NLR* genes showed that 303 (89.6%) of the *NLR* genes were expressed in different varieties. Overall, this study described the full *NLR* profile of the *M. ruthenica* genome to provide an important resource for mining disease-resistant genes and disease-resistant breeding.

## Introduction

Plants are vulnerable to many pathogens during their natural growth, including fungi, bacteria, viruses, and nematodes (Kourelis and van der Hoorn, 2018). These pathogens affect the normal growth, reproduction, and physiological metabolism of plants, in addition to inducing the corresponding diseases. However, as sessile organisms, plants cannot actively avoid these microorganisms and lack a somatic adaptive immune system as is found in animals (Chisholm et al., 2006; Fujita et al., 2006). During their long-term interactions with pathogens, plants have evolved a set of effective self-protection mechanisms to resist pathogen invasion and harm. The innate defense system of plants consists of two main layers. The first layer is plant pattern recognition receptor (PRR) recognition of relatively conserved pathogen/microbe-associated molecular patterns (PAMPs/MAMPs) of the pathogens to stimulate downstream basic defense responses and hinder further pathogen growth. This reaction is termed PAMP/MAMP-triggered immunity (PTI) (Lacombe et al., 2010; Zipfel, 2014). At the same time, pathogens also interfere with the PTI response of host plants and enhance pathogen toxicity through excretive effectors. Effector-triggered immunity (ETI) is another plant immune response that recognizes some effectors directly or indirectly *via* resistance proteins (R proteins) that are encoded by resistance genes (*R* genes) in cells to inhibit further pathogen infection and reproduction (Thomma et al., 2011; Dou and Zhou, 2012). Therefore, *R* genes play an essential role in plant disease resistance processes.

To date, more than 300 *R* genes have been cloned from different plant species since the first *R* gene *Hm1* of maize was cloned in 1992 (Johal and Briggs, 1992). Among them, more than 60% of genes belong to the nucleotide-binding site (NBS)-leucine-rich repeat receptor (LRR) (*NBS-LRR*, *NLR*) gene family (Kourelis and van der Hoorn, 2018). This gene family is the largest disease resistance gene family in plants, and some *NLR* genes are dominant with functions in plant immunity (Ellis and Jones, 1998; Shao et al., 2016; Kourelis and van der Hoorn, 2018). The proteins encoded by *NLR* genes contain three domains: the variable N-terminal domain, central NBS domain, and C-terminal domain LRR (Shi et al., 2018). The NBS domain consists of about 300 amino acid sequences and the C-terminal domain usually has 10–40 short LRR sequences (Traut, 1994; Jones and Jones, 1997). The NBS and LRR domains provide the energy required for signal transduction and recognition of specific pathogens, respectively (Goyal et al., 2020). According to the N-terminal domains, the proteins encoded by *NLR* genes can be further divided into coiled-coil (CC)-NBS-LRR (*CNL*), toll/interleukin-1 receptor (TIR)-NBS-LRR (*TNL*), and resistance to powdery mildew 8 (RPW8)-NBS-LRR (*RNL*) subclasses. Among them, the *CNL* and *TNL* subclasses are commonly used as sensors to detect pathogens, while the *RNL* subclass is used in immune signal transduction (Zhang et al., 2020).

With the publication of plant whole-genome data, *NLR* genes of many plant species [i.e., *Arabidopsis thaliana* (Meyers et al., 2003), rice (Zhou et al., 2004), maize (Cheng et al., 2012), barley (Li et al., 2021), tomato (Liu et al., 2014), potato (Jupe et al., 2012), pea (Djebbi et al., 2015), and alfalfa (Shao et al., 2014)] have been identified and analyzed, which has greatly promoted the understanding of their structures, mechanisms, and evolution. For example, Li et al. (2021) identified *NLR* genes in barely, which contains one *RNL* and 468 *CNL* genes. Qian et al. (2021) identified one *RNL* and 581 *CNL* genes in *Secale cereale*. Similarly, studies have reported no *TNL* subclass in monocotyledons, while both *TNL* and *CNL* subclasses have been found in dicotyledons, and *RNL* has been detected in angiosperms (Andersen et al., 2018; Zhang et al., 2020). In other words, *NLR* genes have differentiated during evolution in different plants. Moreover, *NLR* genes existed before the differentiation of green plants and greatly amplified after plants occupied land (Shao et al., 2019; Liu et al., 2021). The genetic diversity analysis of *NLR* genes based on whole-genome data has not only provided important resources for the exploration of functional disease-resistant genes but also has important value for molecular marker-assisted breeding in plants (Qian et al., 2021). Many genes or resistance gene analogs (RGAs), such as *Rpi-amr3i* from *Solanum americanum* (Witek et al., 2016), *Sm1* from wheat (Walkowiak et al., 2020), and *SRC7* from soybean (Yan et al., 2022), have been cloned by genome-wide *NLR* gene identification. Some of these have become the focus for efforts to improve disease resistance in plants and have been used in agricultural production. *NLR* genes are also widely involved in many other biological processes such as plant growth, development, environmental adaptation, and abiotic stress (Chini et al., 2004; MacQueen and Bergelson, 2016; Ren et al., 2020). Therefore, further studies on *NLR* genes are needed to expand the *NLR* gene database.

*Medicago ruthenica* (L.) (2*n* = 2*x* = 16) is a perennial legume forage grass species and homologous with *M. sativa* (Small and Jomphe, 1989). The latter is “the queen of forage crops” and one of the economically most important forage crops in the world (Brummer, 2004; Liu et al., 2013; Zhou et al., 2019). Because of its strong drought, cold, salt and alkali resistance and abundant leaves, good palatability, and high nutritional quality, *M. ruthenica* has been used not only in the genetic improvement of alfalfa but also as a high-quality pasture to provide nutrition for livestock (Wang et al., 2008; Li et al., 2013). *M. ruthenica* ‘Mengnong No. 1’ was approved for release by the Grass Variety Approval Committee of Inner Mongolia in 2019 (No. 2019003). Our previous study reported its average hay yield of 11,000–15,000 kg/hm^2^ after 3 years of cultivation, 113 first-order branches, and average crude protein content of 11–13% in Tumote, Hohhot, Inner Mongolia. However, like other crops, *M. ruthenica* is also susceptible to a variety of pathogens and diseases, such as powdery mildew, rust disease, and anthracnose. These diseases lead to serious yield and quality losses and further harm the health of livestock (Liu et al., 1989). Therefore, the study of the composition and distribution of *R* genes could help improve the recognition of disease resistance in *M. ruthenica*.

Fortunately, the whole genome of wild *M. ruthenica* has been sequenced and assembled (Wang et al., 2021). Specifically, PacBio, Illumina, 10× Genomics, and Hi-C technologies were used to assemble a 904.13 Mb genome with a scaffold N50 of 99.39 Mb. A total of 49,176 protein-encoding genes were annotated. This genome has provided information for the study of genome-wide *NLR* genes and a foundation for cloning the disease resistance genes of *M. ruthenica*. In this study, we made full use of the whole genome data of *M. ruthenica* to extract the coding sequences (CDSs) of *NLR* genes and analyze the *NLR* genes, including their classification, chromosome localization, conserved amino acid sequence, phylogenetics, duplication type, and synteny analysis. Furthermore, we analyzed the transcriptomes of powdery mildew resistance and susceptible varieties and characterized the expression of these *NLR* genes. The objective of this study was to provide comprehensive information on *NLR* genes in *M. ruthenica*. The results provide a reference for research on *NLR* gene functions and the genetic breeding of *M. ruthenica*.

## Materials and methods

### Data used in this study

The gff3 annotation files and genome sequences of *M. ruthenica* (https://doi.org/10.6084/m9. figshare.12726932) and amino acid sequences and gff3 annotation files of *A. thaliana* (https://www.ncbi.nlm.nih.gov/data-hub/genome/GCF_000001735.4/; https://www.arashare.cn/index/News/info/id/1699.html) and *M. truncatula* (http://plants.ensembl.org/Medicago_truncatula/Info/Index) were downloaded from public databases. Software “gffread” was used to extract the CDS sequences of *M. ruthenica*, and a Perl programming script was used to convert them into amino acid sequences.

### Identification and classification of *NLR* genes in *M. ruthenica*

The *NLR* genes of *M. ruthenica* were identified from the whole genome using hidden Markov models search (HMMsearch) methods. The HMM profile (accession number PF00931) was downloaded from the Pfam database (http://pfam.xfam.org/) (Shao et al., 2014). NLR protein sequences were identified from whole-genome amino acid sequences of *M. ruthenica* using the hmmsearch command with an expectation value (E-value) of ≤0.0001. The obtained protein sequences were extracted using TBtools (Chen et al., 2020). These sequences were then compared to the NLR protein sequence from *A. thaliana*, and those that did not map onto the NLR protein family were eliminated. *A. thaliana NLR* genes were identified and classified based on whole-genome and gff3 files of *A. thaliana*, respectively. The extraction method for the *A. thaliana NLR* genes was consistent with that applied for *M. ruthenica*. Subsequently, the *NLR* genes identified in *M. ruthenica* were renamed according to the naming principles of Ameline-Torregrosa et al. (2008b).

The Pfam database (http://pfam.xfam.org/search) was used to scan for remaining proteins to confirm the presence of the NBS (also named NB-ARC) and TIR domains. Genes that did not encode a conserved NBS domain were removed from the subsequent analyses. The non-redundant candidate sequences were submitted to the NCBI Conserved Domains Database (http://www.ncbi.nlm.nih.gov/Structure/cdd/wrpsb.cgi) to identify the CC, RPW8, LRR, and other integrated domains (IDs). Meanwhile, proteins with no specific domains were compared to the *CNL*, *RNL*, and *TNL* subclasses in *A. thaliana.* Those with intact CC-NBS-LRR, TIR-NBS-LRR, or RPW8-NBS-LRR structures were divided into the corresponding gene subclass. The results from the two methods were combined, and the specific domains were selected for classification when the two results conflicted. Integrated domains with complete domains and E-value <10^−5^ were retained. Finally, the results were saved to Excel 2019 file format and viewed on an online website for data analysis (https://www.hiplot.com.cn/).

### Chromosomal distribution of *NLR* genes in *M. ruthenica* genome

The location information of the *NLR* genes and the conserved regions were extracted using TBtools (Chen et al., 2020). The chromosomal distribution of *M. ruthenica NLR* genes was plotted using an online tool (https://hiplot-academic.com). A sliding window analysis was performed with 1 kb and 250 kb as the step and window sizes, respectively. The principle of clustering was that the distance between two *NLR* genes should be <250 kb on a chromosome (Ameline-Torregrosa et al., 2008b).

### Motif analysis

MEME (https://meme-suite.org/meme/) was used to predict the motifs in the NBS domains of the identified NLR proteins. The maximum motif search value was set at 15, while the other parameters used the default settings (Bailey et al., 2015). The subsequent results of MEME were imported into TBtools for visualization (Chen et al., 2020). Finally, the highly conserved motifs were extracted and compared to reported amino acid sequences to confirm the type of conserved amino acid (Meyers et al., 2003; Ameline-Torregrosa et al., 2008b; Gong et al., 2021).

### Phylogenetic analysis

*M. ruthenica* NLR proteins encoded by typical *NLR* genes with intact CNL, TNL, or RNL domains were selected for sequence alignment and phylogenetic analysis. MEGA 7.0 and ClustalW software were used to align and correct the amino acid sequences. The phylogenetic tree was constructed using Construct/Test Neighbor-Joining Tree in MEGA with a bootstrap test of 500 times. The image was further processed using the online software iTOL (https://itol.embl.de/).

### Synteny and gene duplication analysis

NLR protein amino acid sequences from *M. ruthenica* were used as both a database and query to perform pair-wise all-against-all BLASTp. The results obtained with the gff3 annotation file, including chromosome number, gene identifier, and gene starting and ending positions, were input into MCScanX to classify the types of gene duplication using the duplicate gene classifier program (Wang et al., 2012). Similarly, the file obtained by BLASTp was also used for the analysis of intra-species collinearity using MCScanX software. Circos diagrams were drawn using Circos (v. 0.67; Krzywinski et al., 2009). For cross-species synteny analysis, the NLR proteins of *M. truncatula* (using an extraction method for genomic information consistent with that described for *M. ruthenica*) were used as queries and NLR proteins of *M. ruthenica* were used as a database for pair-wise all-against-all BLASTp. The remaining steps were consistent with the intra-species comparison.

### Expression analysis

Two *M. ruthenica* varieties (‘Zhilixing’ and ‘Mengnong No. 1’) were used for the disease resistance experiment. Seedlings were transplanted in 2019 at Inner Mongolia Agricultural University (111.7°E, 40.8°N), with one plant per hole. The holes in each row were separated by 0.5 m, and the rows were separated by 0.5 m. Three replicates of each variety were set, and each replicate was grown in a 4.0 m × 5.0 m plot. The plants were cultivated using conventional field management. The materials were naturally infected with powdery mildew. The infection types of different *M. ruthenica* varieties were observed and photographed from July to September 2021.

To further characterize the expression patterns of *NLR* genes in different varieties of *M. ruthenica*, leaves of three plants were selected randomly for gRNA extraction and sequencing. The expression analysis was as follows. First, the genes related to disease resistance were selected from the whole-transcriptome data. Then, the *NLR* genes were screened out, and the fragments per kilobase of exon per million reads mapped (FPKM) values representing the expression of each gene in different samples were extracted (Li et al., 2009; Trapnell, C et al., 2012; Kim et al., 2019). Finally, genes with large differential expression in the two samples were selected and submitted to the online data analysis website (https://www.hiplot.com.cn/) to generate a heatmap.

## Results

### Identification and classification of *M. ruthenica NLR* genes

A total of 338 *NLR* genes with high confidence were identified from the sequenced and assembled genome of *M. ruthenica* (Wang et al., 2021). The resulting genes, their corresponding alias names, and original gene identifiers are listed in Supplementary Table S1. *NLR* genes accounted for 0.69% of all annotated protein-coding genes (49176). The obtained *NLR* genes were assigned into three subclasses (*RNL*, *CNL*, and *TNL*) based on the domain component of the protein analyses. These subclasses included 4, 198, and 136 genes, respectively. The specific information on each *NLR* gene, such as gene composition and gene annotation, provided the basic gene resources for the subsequent analyses.

### Domain analysis

**FIGURE 1:**
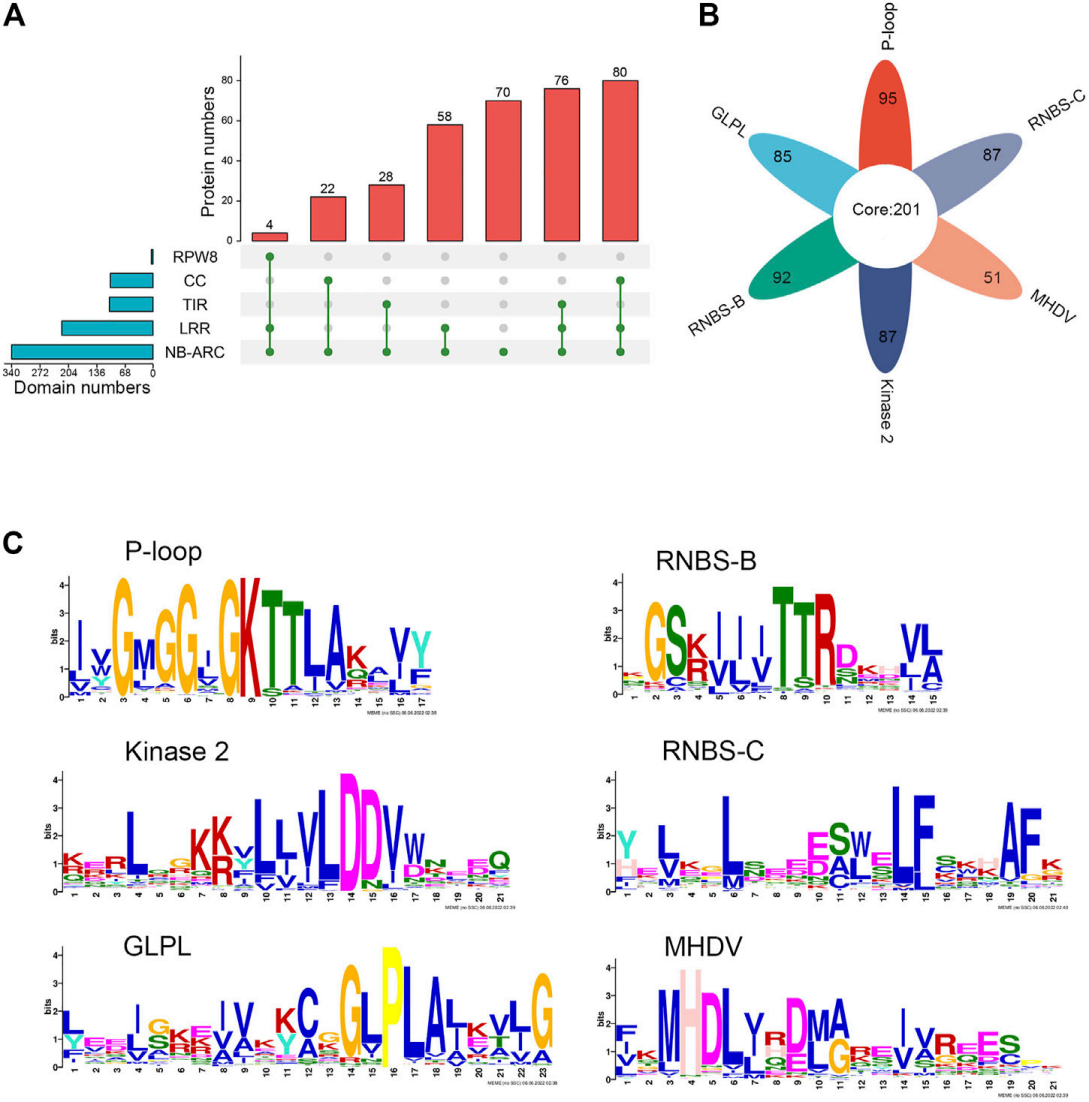
Identification and sequence analysis of *NLR* family genes in *M. ruthenica*. **(A)** Domain compositions of *M. ruthenica* NLR proteins. **(B)** Presence of six key motifs in the amino acid sequence of the NBS domain of *M. ruthenica*. **(C)** Amino acid features of the six key motifs in the amino acid sequence of the NBS domain of *M. ruthenica*.

The protein sequences encoded by the different subclasses of *NLR* genes were further analyzed. The results were visualized according to the type of domain arrangement (Figure 1A; Supplementary Table S1). Among the 198 genes in the *CNL* subclass, 80 encoded intact CNL domains that simultaneously contained the typical N-terminal CC domain, the central NBS domain, and the C-terminal LRR domain, accounting for 40.4% of all *CNL* genes. A total of 22 genes did not encode the LRR domain (i.e., CN), 42 genes did not encode the CC domain (i.e., NL), and 54 genes did not encode either CC or LRR domains (i.e., N) in the *CNL* subclass. Similarly, a total of 76 proteins contained intact domains in the *TNL* subclass, with some atypical *TNL* genes also observed. A total of 28 and 16 TNL proteins did not contain TIR and LRR domains, respectively (i.e., TN or NL), and 16 proteins were missing both LRR and N terminal domains (i.e., N). All four RNL proteins contained intact domains. Among the 338 NLR proteins, four had an RPW8 domain, 104 had a TIR domain, 102 had a CC domain, 218 had an LRR domain, and all had an NBS domain. In general, 338 NLR proteins showed high diversity in their domain arrangements, with 160 (47.3%) typical proteins showing intact domains, and the rest (178) lacking at least one important domain. The ratio of typical CNL, TNL, and RNL proteins was 20:19:1. Some of the 338 NLR proteins also had additional integrated domains, such as PLN03210, PPP1R42, or CDC6.

### Conserved motif analysis

MEME analysis was used to detect the presence of key motifs in the amino acid sequences of the NBS domains. The results revealed six motifs (P-loop, GLPL, RNBS-D, kinase-2, RNBS-C, and MHDV) that were readily detected and highly conserved in *M. ruthenica* NLR proteins, as has been reported for other angiosperms (Meyers et al., 2003; Ameline-Torregrosa, et al., 2008b) (Figures 1B, C). Specifically, 85% of the NLR proteins contained P-loop, GLPL, RNBS-D, kinase-2, and RNBS-C motifs, while 75% of the NLR proteins contained the MHDV motif (Supplementary Table S1). Meanwhile, 201 NLR proteins contained all six motifs simultaneously, accounting for 59.5% of all NLR proteins. Other proteins had lost at least one key motif in the NBS domain. Two NLR proteins did not contain any of the six key motifs, accounting for only 0.59% of the total (Supplementary Table S1). Except for RNBS-B, which was absent in RNL, the remaining motifs were present in most typical RNL, TNL, and CNL proteins, indicating that the RNL protein differed from TNL and CNL in terms of the length and amino acid composition of the conserved sequences. The NLR proteins of *M. ruthenica* also showed some exceptions, including those among the 80 typical CNL proteins, in which one lacked the GLPL motif, one lacked the RNBS-B motif, one lacked the Kinase 2 motif, and eight lacked the MHDV motif. These differences can be used to specifically identify the *NLR* genes of *M. ruthenica* and further distinguish these genes.

### Distributions of *NLR* on the *M. ruthenica* chromosomes

**FIGURE 2:**
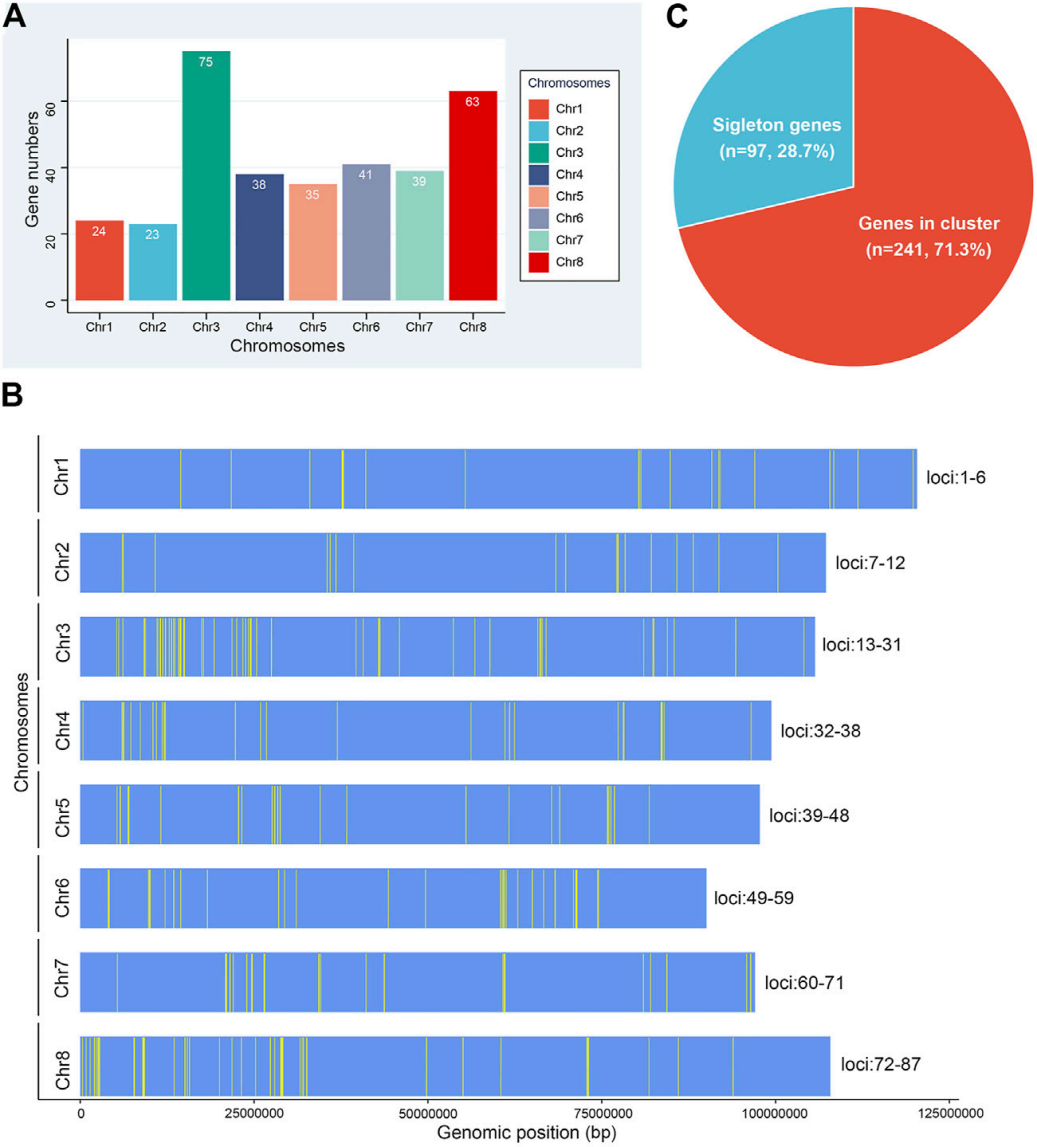
Chromosomal distribution of *NLR* genes in *M. ruthenica*. **(A)** Number of *NLR* genes on the eight chromosomes of *M. ruthenica*. **(B)** Physical locations of *NLR* genes on the eight chromosomes of *M. ruthenica*. **(C)** Proportions of *NLR* genes appearing as singletons (blue) and clusters (red) in the *M. ruthenica* genome.

Gene families dispersed in different positions generally show different patterns of expression regulation and perform important functions. Our analysis of their chromosomal distribution showed that all 338 *NLR* genes of *M. ruthenica* mapped to specific chromosomes (Figure 2A). Among them, the highest number of *NLR* genes mapped to chromosome 3 (75), followed by chromosome 8 (63). In contrast, the number was the lowest on chromosome 2 (23), which was only one less than on chromosome 1 (24). Chromosomes 4 and 7 possessed similar numbers of *NLR* genes (38 and 39). Chromosomes 5 and 6 contained 35 and 41 *NLR* genes, respectively. These results indicated that *NLR* genes were not evenly distributed on the eight chromosomes of *M. ruthenica* and that 138 (40.8%) of the 338 *NLR* genes were mainly distributed on chromosomes 3 and 8.

The physical locations of the 338 *NLR* genes were extracted and further mapped on chromosomes (Figure 2B). The results showed the uneven distribution of these genes and that most were organized into clusters. A total of 87 clusters including 241 (71.3%) *NLR* genes were identified on eight chromosomes (Figure 2C). Chromosome 3 contained the most clusters (19), while chromosomes 1 and 2 had the fewest clusters (6). Three clusters, including seven *NLR* genes each, were considered the largest clusters. Overall, each cluster contained an average of three genes. Most clusters only contained two genes (47), accounting for 54.0% of all clusters. In addition, 97 *NLR* genes existed as singletons, accounting for 28.7% of all *NLR* genes. Overall, the *NLR* genes of *M. ruthenica* mainly existed in clusters, with few singletons. These results indicated that most *NLR* gene clusters of *M. ruthenica* mainly consisted of closely related genes, which shared a common ancestor, similar structure and function, and encoded similar protein products. In addition, the genes existing as singletons most likely evolved independently with new sources of mutation, or had close homologs elsewhere in the genome and might participate in more stable protein complexes or long-term protective functions (Mackey et al., 2003).

### Phylogenetic analysis

**FIGURE 3:**
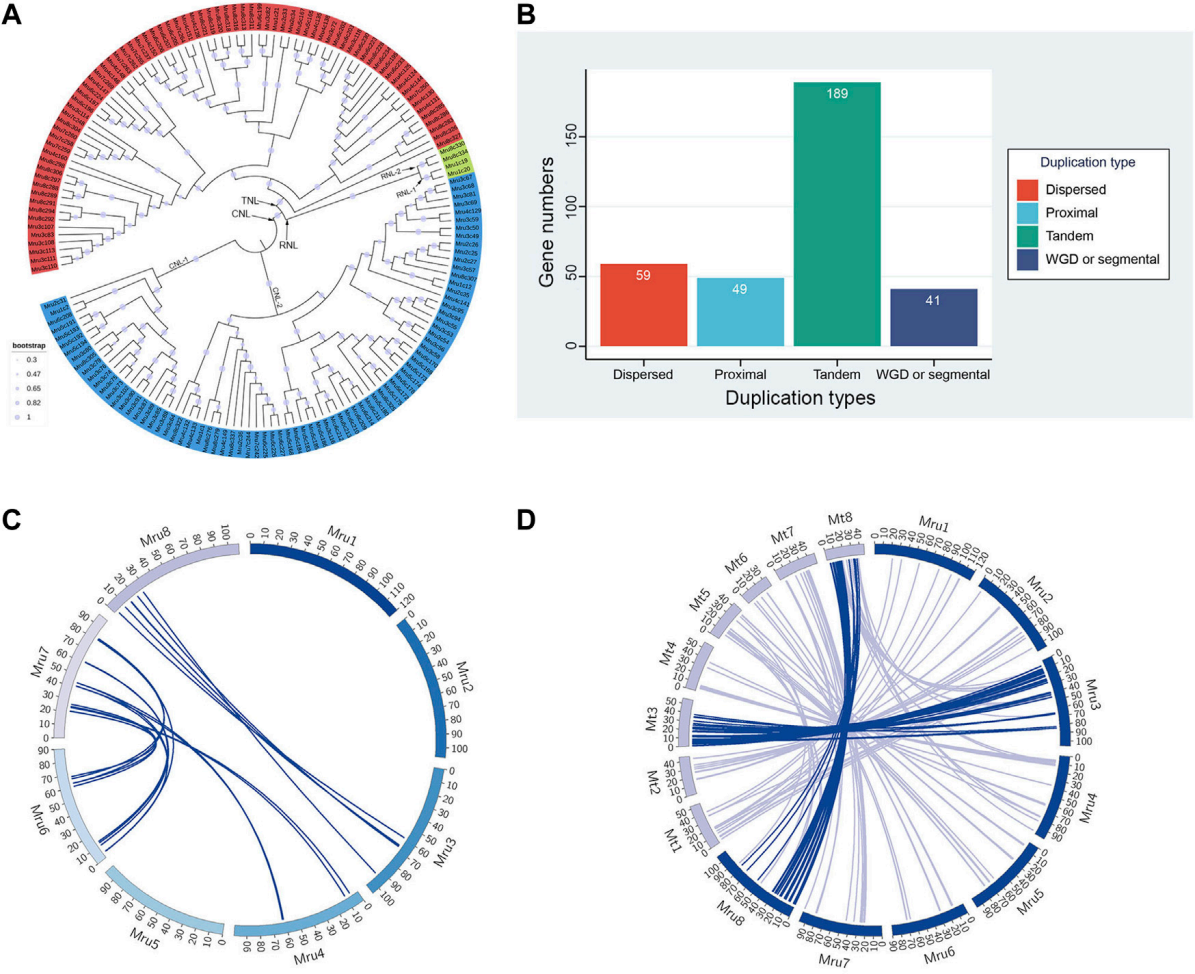
Phylogenetic, gene duplication, and synteny analyses of *NLR* genes in *M. ruthenica*. **(A)** Phylogenetic analysis of typical *NLR* genes in *M. ruthenica*. *RNL*, *CNL*, and *TNL* genes are shown in green, blue, and red, respectively. **(B)** Numbers of each duplication type of *NLR* genes. **(C)** Synteny analysis of *NLR* genes in *M. ruthenica*. **(D)** Synteny analysis of *NLR* genes between *M. ruthenica* and *M. truncatula*.

To understand the separations and evolutionary relationships among the *NLR* genes of *M. ruthenica*, we performed phylogenetic analyses of amino acid sequences of 160 typical *NLR* genes. The results showed that *M. ruthenica NLR* genes formed three deeply separated clades, representing the three major subclasses *RNL*, *TNL*, and *CNL*, respectively (Figure 3A). The four *RNL* genes were further separated into two lineages, namely, RNL-1 and RNL-2. *Mru1c19* and *Mru1c20*, and *Mru8c330* and *Mru8c334* formed highly supported lineages, respectively. The domain analysis showed that RNL-1 contained the integrated domain PLN03210, while RNL-2 did not (Supplementary Table S1).

A total of 80 *CNL* genes in *M. ruthenica* formed two clades, including 21 genes in CNL-1 and 59 genes in CNL-2. CNL-2 was further divided into two or three lineages, which were more closely related. This phenomenon was also observed in the *TNL* clade. Specifically, a total of 76 *TNL* genes in *M. ruthenica* formed two clades, in which the gene *Mru8c327* became a single clade closer to *CNL*, while the other genes belonged to another clade. The possible reasons for this may be related to the PLN03150 domain, which was not detected in other genes (Supplementary Table S1). In general, the evolution of *NLR* genes in *M. ruthenica* was mainly affected by the different types of N-terminal domains. In addition, other integration domains such as PLN03210 and PLN03150 also influenced the genetic relationship between different genes.

### Gene duplication analysis

The duplication types of the 338 *NLR* genes were analyzed using MCScanX (Wang et al., 2012). The results revealed four different duplication types (e.g., tandem array, proximal, dispersed, and segmental duplicate) in *M. ruthenica* (Figure 3B; Supplementary Table S1). The largest group contained 189 (55.9%) tandem array genes. Proximal, dispersed, and segmental duplicate types included 49 (14.5%), 59 (17.5%), and 41 (12.1%) genes, respectively.

### Synteny analysis of *NLR* genes in *M. ruthenica*

Pair-wise all-against-all BLASTp of the 338 *NLR* genes in *M. ruthenica* was used to determine and visualize as circle plots the intra-species genomic synteny (Figure 3C; Supplementary Table S2). Synteny analysis identified 23 *NLR* gene pairs located on syntenic chromosomal blocks. Among the eight chromosomes, the highest number of syntenic *NLR* gene pairs was observed between chromosomes 6 and 7 (12 pairs). In contrast, six and five pairs of syntenic genes were identified between chromosomes 3 and 8 and between chromosomes 4 and 7, respectively. Some genes were associated with two different genes on different chromosomes to form two different syntenic gene pairs. For example, *Mru7c261* and *Mru7c262* located on chromosome 7 were collinear with *Mru4c147* and *Mru4c148* located on chromosome 4, and *Mru6c196* and *Mru6c197* located on chromosome 6, respectively (Supplementary Table S2). Some genes were associated with two different genes on the same chromosome to constitute different syntenic gene pairs. For example, *Mru6c196* and *Mru6c221* were, respectively, collinear with *Mru7c261* and *Mru7c269*, as well as *Mru7c268* and *Mru7c253* located on chromosome 7. In general, the multiple copies of *NLR* genes in *M. ruthenica* were mainly distributed on chromosomes 6 and 7.

To confirm the homology of *NLR* genes between *M. ruthenica* and its related species, the *NLR* genes from *M. truncatula* were extracted for inter-species analysis with pair-wise all-against-all BLASTp (Figure 3D; Supplementary Table S2). Synteny analysis revealed 193 *NLR* gene pairs located on syntenic chromosomal blocks of the two species. Among the eight chromosomes, the highest number (63) of syntenic *NLR* gene pairs was located on chromosome 3. However, chromosome 1 of *M. ruthenica* and chromosome 3 of *M. truncatula* had seven pairs of syntenic *NLR* genes. These results indicated the significant synteny relationship between these chromosomes in *M. ruthenica* and *M. truncatula*.

### Expression analysis of *NLR* genes in *M. ruthenica*

**FIGURE 4:**
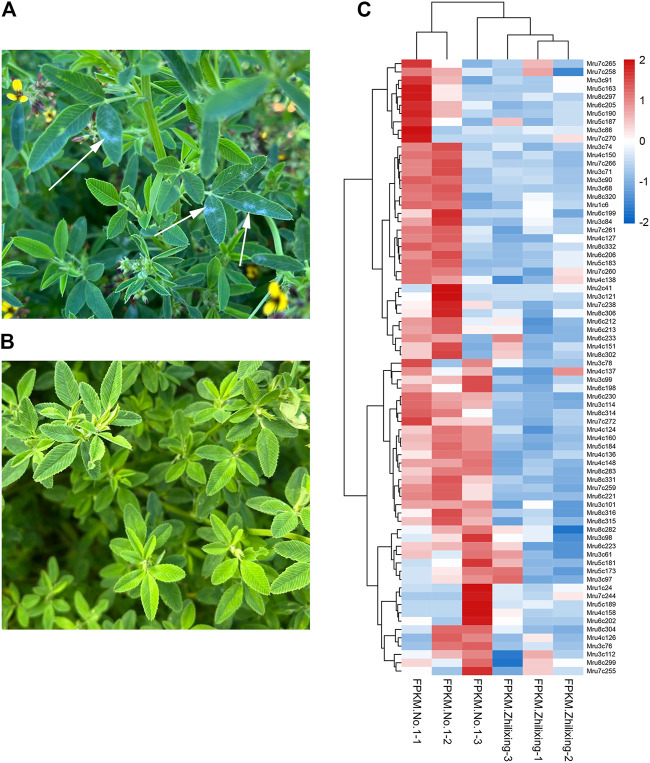
Powdery mildew resistance and expression analysis of different *M. ruthenica* varieties at the initial flowering stage in Inner Mongolia. **(A)** *M. ruthenica* ‘Zhilixing’. Arrows: powdery mildew. **(B)** *M. ruthenica* ‘Mengnong No. 1’. **(C)** Expression heatmap of 72 *NLR* genes differentially expressed between *M. ruthenica* ‘Zhilixing’ and ‘Mengnong No. 1’.

The transcriptome data of *M. ruthenica* ‘Zhilixing’ and *M. ruthenica* ‘Mengnong No. 1’ (Unpublished) were used to characterize the different expression levels of the 338 *NLR* genes. ‘Zhilixing’ is more susceptible to powdery mildew than ‘Mengnong No. 1’ in the initial flowering stage (Figures 4A, B). Overall, 303 of 338 *NLR* genes showed differences in expression levels (Supplementary Table S3). Specifically, 72 *NLR* genes of Mengnong No. 1 showed at least 1.5-fold higher expression than ‘Zhilixing’ (Figure 4C). In addition, 22 genes, including *Mru3c66*, *Mru3c69*, and *Mru8c295*, were not expressed in ‘Zhilixing’ but were expressed or slightly expressed in Mengnong No. 1 (Supplementary Table S3). The expression analysis revealed 303 *NLR* genes with different expression between the two varieties, which may play an important role in *M. ruthenica* powdery mildew resistance responses in the field.

## Discussion

*M. ruthenica*, a relative species of *M. sativa*, is often used as a high-quality genetic resource to improve the abiotic stress resistance of alfalfa and other pasture species because of its high tolerance to various extreme environmental conditions (Campbell et al., 1999; Guan et al., 2009). Therefore, increasing numbers of studies have focused on the response mechanisms, resistance breeding, and the screening and cloning of abiotic stress resistance genes in *M. ruthenica* (Shu et al., 2018; Shi et al., 2021; Yin et al., 2021). However, research on the resistance of *M. ruthenica* to biological stress is lacking, especially in disease resistance breeding and cloning disease resistance genes. The genome-wide identification of the *NLR* gene family would accelerate the process of cloning resistance genes and understanding the corresponding resistance mechanisms (Xue et al., 2020). The genome-wide identification of *NLR* genes in angiosperms has recently been reported. These studies have mainly focused on *NLR* gene numbers, classification, distribution, and conserved domains (Huang et al., 2008; Lozano et al., 2015). The present study is the first to systematically study the *NLR* genes of *M. ruthenica* through bioinformatics analysis based on whole-genome data. The results showed that *M. ruthenica* contains 338 *NLR* genes, 160 of which encode NLR proteins with intact domains, representing approximately 47.3% of the total *NLR* genes. The remaining genes encode only partial domains of NLR proteins with high similarity to domains with full-sized NLR in *A. thaliana*. The number of *NLR* genes in *M. ruthenica* was comparable to that of *M. truncatula* A17 and common bean. Specifically, *M. truncatula* A17 has 333 *NLR* genes containing 177 *CNL* and 156 *TNL*, while the common bean has 337 *NLR* genes containing 103 *TNL*, 224 *CNL*, and 10 *RNL* (Ameline-Torregrosa et al., 2008b; Shao et al., 2014). The total number of *NLR* genes in *M. ruthenica* represented 0.69% of the total annotated genes, which is consistent with the proportions (0.6%–1.76%) of *NLR* genes in other plants (Porter et al., 2009). Thus, although the *NLR* genes evolved rapidly, they have largely not been lost in *M. ruthenica*.

The NLR proteins contained typical domains with highly similar sequences, which are an important component of NLR proteins and have important functions in disease resistance (Bai et al., 2002). Among the three major domains (TIR/CC/RPW8, NBS, and LRR), NBS is the core domain of NLR proteins and is present in G-protein and GTP/ATP-binding protein families, which bind and hydrolyze ATP and GTP and participate in the nucleoside triphosphate metabolic pathway (Downward, 1990; Bourne et al., 1991; Kaziro et al., 1991; Traut, 1994; Tameling et al., 2002; Rojas et al., 2012). Several studies have reported that the LRR domain specifically recognizes pathogens through protein–protein interactions (Kobe and Deisenhofer, 1995; Ellis and Jones, 1998; Leister and Katagiri, 2000; Hulbert et al., 2001; Belkhadir et al., 2004). Moreover, differences in LRR number are the main factor of variation in *NLR* gene length (Jones and Jones, 1997). Mutations in the LRR region confer disease resistance in *A. thaliana* (Bent et al., 1994; Grant et al., 1995; Warren et al., 1998; Gassmann et al., 1999) and rice (Bryan et al., 2000). TIR and CC structures stimulate downstream signaling systems in EDS1 and NPR1 types after *R* gene and pathogen recognition and participate in plant immune response (Medzhitov et al., 1997; Ellis and Jones, 1998; Bai et al., 2002; Monosi et al., 2004). Finally, RPW8 has demonstrated broad-spectrum resistance to mildew in *A. thaliana* (Xiao et al., 2001).

Each domain or variety of domain arrangements of NLR proteins performs different functions and plays an important role in defending against pathogen invasion (Ameline-Torregrosa et al., 2008b). Therefore, the structural domains of NLR proteins were analyzed. Among the proteins encoded by 338 *NLR* genes of *M. ruthenica*, four had the RPW8 domain, 102 had the CC structure, 104 had the TIR domain, 218 contained the LRR domain, and all contained the NBS domain. Overall, 108 of the 338 NLR proteins contained two domains simultaneously, including 58 that contained NL, 22 that contained CN, and 28 that contained TN. Similar various atypical domains have been observed in *M. truncatula* (Ameline-Torregrosa et al., 2008b). In addition, the 338 *NLR* genes of *M. ruthenica* included 160 typical *NLR* genes with intact CNL (80), TNL (76), or RNL (4) structures. This result differs from that of many other plants. For example, the ratios of *CNL* and *TNL* genes were nearly 1:2 in *A. thaliana* and potato and 1:3 in grape (Meyers et al., 2003; Lozano et al., 2012; The Tomato Genome Consortium, 2012). Moreover, no *TNL* genes have been detected in many Gramineous, and only a minority of *RNL* genes have been found (Cheng et al., 2012; The Tomato Genome Consortium, 2012; Li et al., 2021; Qian et al., 2021). However, in our study, the ratio of typical *CNL* and *TNL* was close to 1:1, with slight differences in the numbers of genes in the *CNL* (198) and *TNL* (136) subclasses. The result was similar for *M. truncatula* whose *CNL*, *TNL*, and *RNL* genes accounted for 48.9%, 48.1%, and 3% of these genes, respectively (Shao et al., 2016). These results suggested that *CNL* and *TNL* played equally important roles in disease resistance in *Medicago* (Leister, 2004; Jupe et al., 2012).

Phylogenetic analysis has been used to show the relationship between different individuals (Kumar et al., 2016). Phylogenetic analyses of *NLR* genes have shown that non-*TNL* (lacking the TIR domain, usually known as *CNL* and *RNL* subclasses) were more diverse than *TNL* genes. Many studies have also suggested that non-*TNL* subclasses are older than *TNL* subclasses (Cannon et al., 2002; Meyers et al., 2002; 2003; Mun et al., 2009). The *TNL* and *CNL* subclasses were first identified among land plants in *Physcomitrella patens* and *Selaginella moellendorffii*, respectively (Yue et al., 2012). Subsequently, the *R* genes of these two subclasses were extended to higher plants and the RPW8 domain has recently been identified in angiosperms (Zhang et al., 2020). However, genes with RPW8 structure were assigned to the *CNL* lineage in some phylogenetic analyses, indicating a closer evolutionary relationship among the genes in these two subclasses (Ameline-Torregrosa et al., 2008b; Lozano et al., 2015). In contrast, in our study, instead of being assigned to the *CNL* subclasses, genes encoding the RPW8 domain in their N-terminal region formed a strong branch distinguished from *CNL* in *M. ruthenica*. This showed that the *RNL* clade was not classified into the *CNL* clade but rather formed an independent monophyletic clade with *TNL* and *CNL*. This finding has also been observed in other legumes, including *M. truncatula*, pigeon pea, soybean, and common bean (Shao et al., 2014). Meyers et al. (2003) also reported that *RNL* genes formed a separate lineage group (CNL-A group) in *A. thaliana* and that its principle of action also differed from that of most *CNL* genes. In addition, *CNL*, *TNL*, and *RNL* genes continued to be divided into smaller clades due to different integration domains. In short, the evolutionary relationships of the *NLR* gene family in *M. ruthenica* are mainly affected by the CC, TIR, and RPW8 domains and other accessory domains. Moreover, the function and phylogeny of *RNL* genes differed significantly between the *CNL* and *TNL* clades in *M. ruthenica*.

Polyploidy and multiple forms of gene doubling increase the copy numbers of homologous genes. Gene duplication is one of the most important evolutionary processes, which generates genetic diversity and new functions, and plays a crucial role in adaptation and speciation (Magadum et al., 2013). In this study, we identified 189, 49, 59, and 41 tandem, proximal, dispersed, and segmental duplicate genes, accounting for 55.9%, 14.5%, 17.5%, and 12.1% of all *NLR* genes, respectively. These results were consistent with the findings of Duan et al. (2019) who performed phylogenetic analysis and reported a large number of tandem duplications of *NLR* genes in legumes, including *Glycine max*, *Arachis duranensis*, and *M. truncatula*. Thus, tandem duplications may have occurred before the doubling of legumes and may have been conducive to the expansion of family gene copy numbers (Ratnaparkhe et al., 2011).

Syntenic analysis could explain the conservation of gene types and the relative order among different species diverging from the same ancestry. A large amount of homology information is contained in syntenic chromosome blocks at the DNA level between related species (Wang et al., 2012). Intra-species syntenic analysis could be used to confirm the homology of different chromosomes and analyze the duplication of regions or genes with multiple copies, while inter-species analysis could show genome homology (Qian et al., 2021). The syntenic analysis of *NLR* genes in *M. ruthenica* revealed 23 *NLR* gene pairs located on syntenic chromosomal blocks associated with chromosomes 6 and 7, 3 and 8, and 4 and 7. The same genes may have different homologs on different chromosomes, indicating that the *NLR* genes of *M. ruthenica* have multiple copies in different directions.

*M. truncatula* (2*n* = 2*x* = 16), a member of the *Medicago* genus, has been used as a model species of legume (Barker et al., 1990; Cook, 1999). A17-Jemalong is one genotype of *M. truncatula* with high resistance to many diseases such as anthracnose and powdery mildew (Ameline-Torregrosa et al., 2008a). Therefore, it was selected as the target species for syntenic analysis with *M. ruthenica*. The results revealed 193 *NLR* gene pairs located on syntenic chromosomal blocks of chromosome 3 between *M. ruthenica* and *M. truncatula*, indicating that *NLR* genes expanded similarly in these two species (Qian et al., 2021). However, earlier studies demonstrated significant loss and rearrangement in the *M. truncatula* genome, especially the *NLR* genes, which suggested rapid evolution and may have eliminated any evidence of syntropy (Ameline-Torregrosa et al., 2008b). Overall, the *NLR* gene family is a large multi-gene family with a complex gene structure and evolutionary process. More comprehensive and detailed analyses are required to better support breeding for disease resistance.

*NLR* gene expression patterns have been reported for many plants, which provide more evidence to understand *NLR* gene structures, functions, and applications (Lowe et al., 2017). Yu et al. (2021) reported that *NLR* gene expression levels in *Akebia trifoliata* were higher in rind tissue that was vulnerable to pathogens. Similarly, Ren et al. (2020) also observed significantly higher *NLR* gene expression in orchardgrass with high resistance to rust fungus compared to the expression in highly sensitive materials. The same results were reported in other plants, including *Cucumis sativus* (Zhang et al., 2022), *Helianthus annuus* (Neupane et al., 2018), *Hordeum vulgare* (Wang et al., 2013), and *Populus* (Bresson et al., 2011). In the present study, we found that 303 (89.6%) *NLR* genes were expressed in different *M. ruthenica* leaf tissues. Most showed no significant differences in expression, possibly due to the low sequencing coverage or pseudogenization (Frazier et al., 2016; Neupane et al., 2018). However, 72 of 303 *NLR* genes showed higher expression in resistant varieties compared to susceptible varieties, which indicated that these *NLR* genes might play important roles in the resistance of *M. ruthenica* to powdery mildew and may provide new resistance sources for the improvement of *M. ruthenica* disease resistance, although their specific functions and regulatory mechanisms are still unclear. Additional study on the functions of *NLR* genes of *M. ruthenica* under abiotic stress conditions is needed. The results of the present study have laid the foundation for further understanding. In general, *NLR* genes in *M. ruthenica* play important roles in pathogen defense to guarantee the normal growth and development of *M. ruthenica* in different ecological environments.

## Conclusion

Analysis of whole-genome data of *M. ruthenica* uncovered a draft *NLR* gene family, which included 338 *NLR* genes with high confidence and diversity. These genes can be divided into three subclasses (*TNL*, *CNL*, and *RNL*) according to their domain arrangements. Six motifs (P-loop, GLPL, RNBS-D, kinase-2, RNBS-C, and MHDV) exist in the amino acid sequences of the NBS domains. The *NLR* genes of *M. ruthenica* mainly mapped to chromosomes 3 and 8, and most were organized in gene clusters. Phylogeny analysis revealed that genes in the *TNL*, *CNL*, and *RNL* subclasses formed three deeply separated clades. Collinearity analysis showed that the *NLR* genes of *M. ruthenica* had four duplication types, with tandem array genes the most numerous. Moreover, segmental duplication genes formed 23 *NLR* gene pairs located on syntenic chromosomal blocks of *M. ruthenica*. Meanwhile, 193 syntenic *NLR* gene pairs were identified between *M. ruthenica* and *M. truncatula*. Differential expression analysis showed that 303 (89.6%) *NLR* genes could play essential roles in powdery mildew resistance. These results further refine and expand information about *M. ruthenica* disease resistance gene families, which could lay a foundation for screening and cloning disease resistance genes and molecular breeding of disease resistance in *M. ruthenica* and its related species.

## Data Availability

The original contributions presented in the study are included in the article/Supplementary Material; further inquiries can be directed to the corresponding author.
